# The efficacy and safety of conventional transcatheter arterial chemoembolization combined with PD-1 inhibitor and anti-angiogenesis tyrosine kinase inhibitor treatment for patients with unresectable hepatocellular carcinoma: a real-world comparative study

**DOI:** 10.3389/fonc.2022.941068

**Published:** 2022-09-29

**Authors:** Zheng Guo, Huabin Zhu, Xiufang Zhang, Li Huang, Xiangcai Wang, Huaqiu Shi, Li Yu, Yingwei Qiu, Fuping Tu

**Affiliations:** ^1^ Department of Hematology and Oncology, International Cancer Center, Shenzhen Key Laboratory, Hematology Institution of Shenzhen University, Shenzhen University General Hospital, Shenzhen University Health Science Center, Shenzhen University, Shenzhen, China; ^2^ First school of clinical medicine, Gannan Medical University, Ganzhou, China; ^3^ Department of Oncology, First Affiliated Hospital of Gannan Medical University, Ganzhou, China; ^4^ Department of Radiology, Huazhong University of Science and Technology Union Shenzhen Hospital, Shenzhen, China

**Keywords:** unresectable hepatocellular carcinoma, conventional transcatheter arterial chemoembolization, PD-1 inhibitor, tyrosine kinase inhibitor, combination

## Abstract

**Aim:**

We sought to evaluate the efficacy and safety of conventional transcatheter arterial chemoembolization (cTACE) sequentially combined with systemic treatment by programmed cell death protein 1 (PD-1) inhibitor and anti-angiogenesis tyrosine kinase inhibitor (Anti-angiogenesis TKI) in patients with unresectable hepatocellular carcinoma (HCC).

**Materials and methods:**

One hundred and forty-seven advanced HCC patients who received PD-1 inhibitors and TKIs as first-line systemic treatment between August 2019 and April 2021 were collected retrospectively. Fifty-four patients were finally included and divided into cTACE and no-cTACE groups, according to whether cTACE treatment was performed within 8 weeks before systemic treatment. The tumor objective response ratio (ORR), progression-free survival (PFS), overall survival (OS), and adverse events (AEs) were compared between the groups. Significant factors affecting PFS and OS were determined by Cox regression.

**Results:**

Thirty-one patients received cTACE followed by systemic treatment and 23 patients received systemic treatment only. The ORRs of the cTACE group were 48.4% (after two cycles of systemic treatment) and 51.6% (after four cycles of systemic treatment), while those of the no-cTACE group were only 17.4% and 21.7%. cTACE patients also had a longer median PFS (11.70 vs. 4.00 months, *P* = 0.031) and median OS (19.80 vs. 11.6 months, *P* = 0.006) than no-cTACE patients. Regression analyses indicated that cTACE therapy and Eastern Cooperative Oncology Group performance status were independent risk factors for PFS and OS. AEs by type were similar between the cTACE and no-cTACE groups, except for liver function injury, which was more common among cTACE patients. Fourteen patients suffered with grade 1-2 of rash in 21 patients with objective response, while only 10 patients suffered with rash in 33 patients without objective response, the adjusted hazard ratio (HR) was 4.382 (1.297–14.803).

**Conclusions:**

The combination of cTACE and PD-1 inhibitors and anti-angiogenesis TKIs as therapy significantly improved markers of treatment efficacy, including ORR, PFS, and OS, in unresectable HCC patients, while no more serious AEs recorded in this population compared to those receiving systemic treatment alone. Skin rash might be a predict factor to the efficacy of PD-1 inhibitors and TKI treatment.

## Highlights

- cTACE combined with PD-1 inhibitors and TKIs showed more benefits compared to the use of PD-1 inhibitors and TKIs only in unresectable HCC patients.
- No more serious adverse events occurred in HCC patients receiving TACE and systemic therapy compared to systemic therapy only.
- Skin rash may predict the efficacy of systemic therapy in advanced HCC patients.

## Introduction

Hepatocellular carcinoma (HCC) is a common cancer and a leading cause of cancer-related death. Most HCC patients in China are infected with hepatitis B virus (HBV) (1). In the last several decades, effective systemic treatments for unresectable advanced HCC have remained limited. The multi-tyrosine kinase inhibitors (mTKIs) Sorafinib (2) and Lenvatinib (3) have shown low objective response rates (ORRs) and limited survival benefits. Although immune checkpoint inhibitors (ICIs) have achieved good efficacy in many solid tumors, no ICI monotherapy has received priority recommendations for HCC until now (4). ORRs for ICI monotherapies range from only 15%–23% but could be increased to approximately 30% after combination with TKIs or another ICI (5). Anti-angiogenesis medications, including TKIs and mono-antibodies, target vascular endothelial growth factor (VEGF) signaling, normalize tumor blood vessels, regulate the tumor microenvironment, and enhance the efficacy of ICIs (6, 7). Based on the IMbrave-150 (8) and ORIENT-32 (9) randomized controlled trials (RCTs), ICIs and anti-angiogenesis therapeutics in combination are recommended as first-line systemic treatments in advanced HCC patients at present. RESCUE, as a multicenter clinical study, using Camrelizumab and Apatinib as first-line or second-line treatment in advanced HCC also indicated its efficacy and manageable safety in China (10).

Transcatheter arterial chemoembolization (TACE) has been recommended as a primary locoregional treatment for intermediate-stage HCC patients for decades in guidelines of the National Comprehensive Cancer Network (NCCN) and the Chinese Society of Clinical Oncology (CSCO) given its broad range of indications and high short-term effect (11). Based on the embolic materials used, TACE includes conventional TACE (cTACE), which is performed by injecting anti-cancer drugs and lipiodol followed by embolic materials, and drug-eluting bead TACE (DEB-TACE) (12). DEB-TACE has a better treatment response without a greater survival benefit compared to cTACE, but the cost of DEB-TACE is also higher than that of cTACE (13–15). TACE-induced hypoxia in tumor leads to the overexpression of VEGF (16) and activate the proliferation of tumor cells (17), making the combination of TACE and anti-angiogenesis effective in theory. However, the use of anti-angiogenesis in combination with TACE is still limited given the different definitions of median PFS (time-to-unTACEable progression) considered in its few trials (18, 19), which were much longer than that in a previous RCT (20). Furthermore, TACE primarily causes tumor necrosis by embolizing tumor blood supply arteries, and the necrosis of tumor cells leads to the release of neo-antigens and the activation of dendritic cells to the tumor microenvironment, which can change an immunosuppressive microenvironment into an immuno-supportive setting (20), ensuring an improved response to ICIs.

Given the currently available evidence, TACE may be an ideal partner for ICI-based systemic therapy in advanced HCC. To date, clinical studies, such as NCT04246177 (21), NCT04472767, and NCT04997850 (https://clinicaltrials.gov/), are still ongoing to verify the efficacy of TACE combined with TKIs and ICIs. PD-1 inhibitors are the main class of ICI widely used in China in light of their good accessibility and low price. Our study investigates the efficacy and safety of cTACE sequentially with PD-1 inhibitors and TKI treatment for unresectable HCC patients in the real world, which might be helpful for clinical decision-making in practice.

## Methods

### Study design

Data from 147 advanced HCC patients who received PD-1 inhibitors and TKIs as first-line systemic treatment between August 2019 and April 2021 at the First Affiliated Hospital of Gannan Medical University were collected. The institutional committee of the hospital approved the proposal of this study, and the need for written informed consent was waived. All patients were diagnosed and treated according to the guidelines of the Barcelona Clinic Liver Cancer (BCLC) staging system as either Child–Pugh class A or B cases. Patients without surgical indications or who refused to operate was included. cTACE was the most common local treatment in these patients (77/147), in order to investigate the role of cTACE in combined with Camrelizumab, (a PD-1 inhibitor, Hengrui Medicine Co., Ltd., Jiangsu, China) and TKIs, the exclusion criteria were as follows: 1) ablation or radiotherapy was used before systemic treatment; 2) HCC was combined with other malignant tumors; 3) < 2 cycles of ICI were used; 4) the interval time between cTACE (if administered) and systemic treatment was > 8 weeks; 5) the patient was lost to follow-up. Nighty-three patients were excluded followed by the criteria above and the enrolled fifty-four patients were included and divided into a cTACE group (n = 31) and a no-cTACE group (n = 23) according to whether cTACE was performed or not before systemic treatment with PD-1 inhibitors and TKIs.

### Clinical features

Baseline characteristics at diagnosis were collected, including age, sex, Eastern Cooperative Oncology Group (ECOG) performance status, Hepatitis B surface antigen (HBs–Ag) status, Alpha fetoprotein (AFP), Child–Pugh class, BCLC stage, largest tumor size, number of liver tumors, macro vascular invasion, extrahepatic metastasis, and the type of TKI used. Extrahepatic metastasis was evaluated by chest CT scan, enhanced abdominal CT or MRI. The location of extrahepatic metastases in these patients included lung and lymph node. Camrelizumab was administered at the dosage of 200 mg every 3 weeks, the first treatment time of Camrelizumab in cTACE group was within 8 weeks post TACE. For treatment with TKIs, patients received the initial dosage of Sorafenib (400mg bid), Lenvatinib (body weight < 60 kg with 8mg daily, body weight ≥ 60 kg with 12mg daily), or Apatinib (250mg daily), and dose adjustments were made according to the grade of treatment-related adverse events (TRAEs). Routine blood test values, liver function test results, and imaging data from different time points were saved.

### The conventional TACE procedure

Two interventional radiologists with > 10 years of experience performed cTACE using an angiography X-ray system (UNIQ FD20; Philips, Eindhoven, Netherlands). Seldinger method was used for percutaneous arterial puncture, a short guide wire was used to place the catheter sheath, and then the intubation operation was performed under X-ray system. The catheter was super-selectively inserted into the tumor-feeding artery, and chemoembolization was performed with iodized oil (5–20 mL) mixed with platinum (10–40 mg) or epirubicin (10–40 mg). Additional gelatin sponge was used to further enhance embolization in 22 patients without serious liver cirrhosis. After chemoembolization, the feeding vessels of the tumor were blocked, and angiography revealed that tumor staining decreased or disappeared. Pull out the catheter post operation, pressed the puncture site, braked the puncture side limb for 12 hours and lied flat for 24 hours. Reoperation is feasible post 4 weeks of last surgery if necessary.

### Assessments

The modified Response Evaluation Criteria in Solid Tumors (mRECIST) were used by a radiologist with > 10 years of experience to evaluate HCC tumor responses. The criteria to define the response were as follows: 1) complete response (CR), the disappearance of any intra-tumoral arterial enhancement in all target lesions; 2) partial response (PR), ≥ 30% decrease in the sum of enhanced diameters of viable target lesions; 3) stable disease (SD), the total diameter of the target lesion was not reduced and not increased as other grades; and 4) progressive disease (PD), an increase of ≥ 20% in the sum of the diameters of viable target lesions or new lesion/lesions was defined. The target lesions in current study were those with the largest tumor diameter and typical image characteristics and the numbers of target lesion were less than two (22). The percentage of patients with CR plus PR was defined by the ORR, and the percentage of patients with CR, PR, and SD was defined by the disease control rate (DCR). Progression-free survival (PFS) was the time interval from our treatment to the first PD or death, while overall survival (OS) was the time from our treatment to death. TRAEs were graded according to the Common Terminology Criteria for Adverse Events (CTCAE) version 5.0.

### Statistical analysis

Study data were analyzed using the SPSS version 22.0 statistical software (IBM Corporation, Armonk, NY, USA). The chi-squared test was used to detect the differences in clinical features between the cTACE group and no-cTACE group. A waterfall plot revealed the effective depth of treatment in different groups. Logistic analysis was performed to select the related clinical factors for ORR. Cox regression and Kaplan–Meier analysis were used to evaluate the risk factors affecting prognosis, including PFS and OS. The follow-up time for patients in this study was until March 31, 2022 and the median follow-up time was 11.70 months. The sequential treatment post progression of disease in two groups was statistically analyzed by chi-squared test. *P* < 0.05 indicated a statistically significant difference.

## Results

### Patients’ characteristics

**Figure 1:**
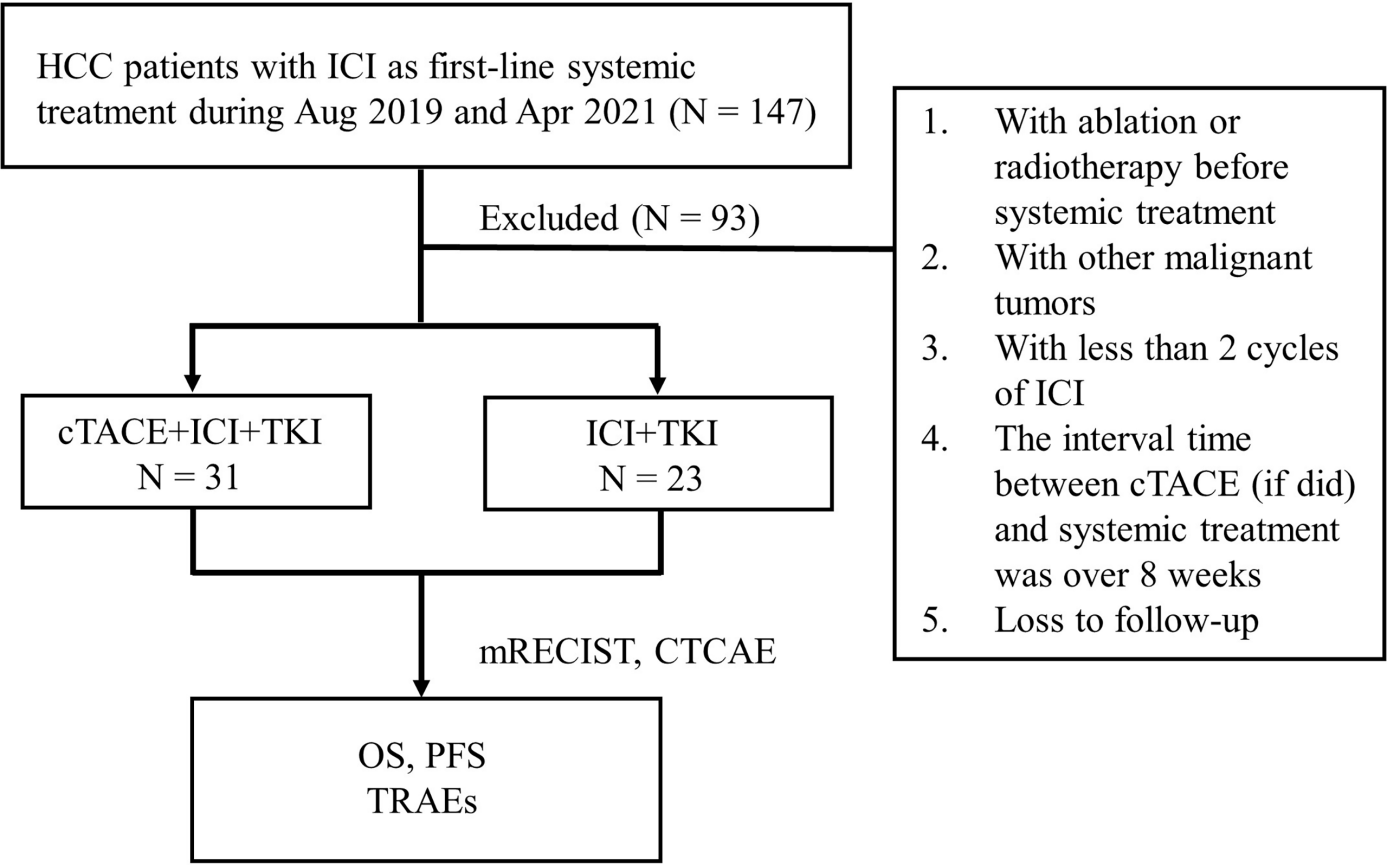
Flowchart of this study.

Fifty-four patients were enrolled and divided into 2 groups as shown in **Figure 1**. The comparison of baseline characteristics, including sex, age, ECOG performance status, HBsAg status, AFP level, Child–Pugh class, largest tumor size, BCLC stage, number of liver tumors, and macro vascular invasion, between the cTACE group (N = 31) and no-cTACE group (N = 23) is presented in **Table 1**. No significant differences were found in these factors between the groups. Three patients with BCLC stage A included in this study experienced recurrence after their operation and could not be operated on. The frequency of cTACE treatment in cTACE group was from 1 to 4 times before the progression of disease, the average of times was 1.36. The proportion of PFS event was 57.4% (31/54), the proportion of OS event was 46.3% (25/54).

**Table 1:** Baseline characteristics of patients before treatment in both groups.

Groups Clinical Features	cTACE (n = 31)	No-cTACE (n = 23)	*P* value
**Sex**			0.355
**Male**	26 (83.9%)	22 (95.7%)	
**Female**	5 (16.1%)	1 (4.3%)	
**Age, years**			0.052
** < 60**	24 (77.4%)	12 (52.2%)	
**≥ 60**	7 (22.6%)	11 (47.8%)	
**ECOG performance status**			0.653
**0 – 1**	22 (71.0%)	15 (65.2%)	
** 2**	9 (29.0%)	8 (34.8%)	
**HBs-Ag**			0.725
(+)	29 (93.5%)	20 (87.0%)	
(−)	2 (6.5%)	3 (13.0%)	
**AFP (ng/mL)**			0.846
**<400**	17 (54.8%)	12 (52.2%)	
**≥400**	14 (45.2%)	11 (47.8%)	
**Child-Pugh class**			0.601
**A (5 – 6)**	21 (67.7%)	14 (60.9%)	
**B (7 – 9)**	10 (32.3%)	9 (39.1%)	
**Largest tumor size (cm)**			0.556
**< 5**	6 (19.4%)	7 (30.4%)	
**≥ 5**	25 (80.6%)	16 (69.6%)	
**BCLC stage**			0.888
**A**	2 (6.5%)	1 (4.3%)	
**B**	5 (16.1%)	3 (13.1%)	
**C**	24 (77.4%)	19 (82.6%)	
**Liver tumor number**			0.846
**<3**	10 (32.3%)	8 (34.8%)	
**≥3**	21 (67.7%)	15 (65.2%)	
**Macro vascular invasion**			0.220
**(+)**	20 (64.5%)	11 (47.8%)	
**(−)**	11 (35.5%)	12 (52.2%)	
**Extrahepatic metastasis**			0.658
**(+)**	17 (54.8%)	14 (60.9%)	
**(−)**	14 (45.2%)	9 (39.1%)	
**TKI**			0.071
**Sorafenib**	11 (35.5%)	12 (52.2%)	
**Lenvatinib**	13 (41.9%)	3 (13.0%)	
**Apatinib**	7 (22.6%)	8 (34.8%)	

+, positive; -, negative.

### cTACE increased the efficacy of PD-1 inhibitors and TKIs

**Figure 2:**
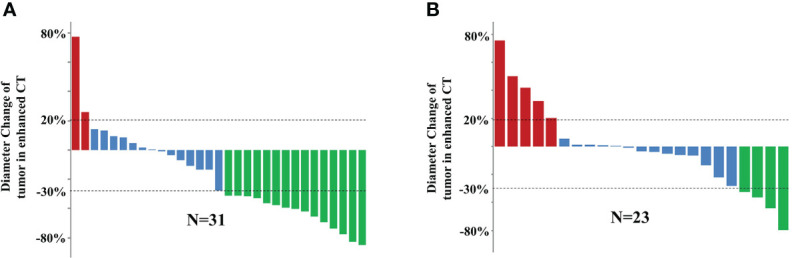
Efficacy of 54 HCC patients with two cycles of systemic treatment in the **(A)** cTACE group and **(B)** no-cTACE group. Red represents progressive disease, blue represents stable disease, and green represents partial response.

**Figure 3:**
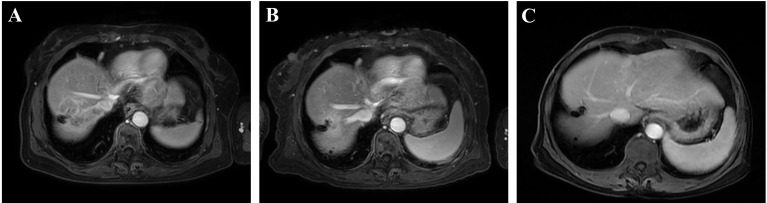
A representative case with complete response in the cTACE group. **(A)** Before treatment; **(B)** after two cycles of systemic treatment; and **(C)** after four cycles of systemic treatment.

The therapeutic efficacy was evaluated using the mRECIST criteria after two and four cycles of PD-1 inhibitor-based systemic treatment, respectively. The ORR in the cTACE group was 48.4% at the first assessment compared to 17.4% in the no-cTACE group (**Table 2a**). The release depth of each patient indicated that a better release of cTACE treatment occurred compared to no-cTACE treatment (**Figure 2**). With a total of four cycles of systemic treatment, the ORRs were 51.6% and 21.7% in the cTACE group and the no-cTACE group, respectively (**Table 2b**). For the significant difference in the number of patients taking Lenvatinib in the combined TACE group and the non-combined group (13 cases versus 3 cases), we performed additional analysis using cases who did not used Lenvatinib, the ORRs of four cycles treatment between two groups was 55.56% (10/18) and 25.00% (5/20), which kept consistent with the primary result although without significant difference (**Table S1**). The cTACE combination improved the ORR by about 30% compared to the no-TACE treatment, while no significant difference was found in DCR between the groups. Two patients in the cTACE group showed CR after four cycles of ICIs and TKIs; **Figure 3** presents images from one of these cases. Furthermore, TACE treatment is defined as an independent factor for short efficacy by logistic analysis in **Table 3**.

**Table 2:** Efficacy of HCC patients with different treatment combinations (with two cycles of ICI).

Groups Efficacy	cTACE (n = 31)	No-cTACE (n = 23)	*P* value
**2a**			
**PR**	15 (48.4%)	4 (17.4%)	0.026*
**SD**	13 (41.9%)	12 (52.2%)	
**PD**	3 (9.7%)	7 (30.4%)	
**ORR (CR + PR)**			0.018*
**(+)**	15 (48.4%)	4 (17.4%)	
**(−)**	16 (51.6%)	19 (82.6%)	
**DCR (CR + PR + SD)**			0.112
**(+)**	28 (90.3%)	16 (69.6%)	
**(−)**	3 (9.7%)	7 (30.4%)	
**2b**			
**CR**	2 (6.5%)	0 (0.0%)	—
**PR**	14 (45.2%)	5 (21.7%)	
**SD**	12 (38.7%)	10 (43.5%)	
**PD**	3 (9.6%)	8 (34.8%)	
**ORR (CR + PR)**			0.026*
**(+)**	16 (51.6%)	5 (21.7%)	
**(−)**	15 (48.4%)	18 (78.3%)	
**DCR (CR + PR + SD)**			0.054
**(+)**	28 (90.3%)	15 (65.2%)	
**(−)**	3 (9.7%)	8 (34.8%)	

*p < 0.05.

**Table 3:** Logistic analysis of clinical factors for ORR (with two cycles of ICI) in 54 HCC patients.

Clinical Factors	Univariable	*P* value
	HR (95% CI)	
**Sex (Male, Female)**	2.000 (0.362–11.048)	0.427
**Age (< 60 y, ≥ 60 y)**	2.600 (0.801–8.437)	0.112
**ECOG performance status (0-1, 2)**	0.451 (0.123–1.654)	0.230
**HBs–Ag (+, −)**	0.323 (0.049–2.131)	0.241
**AFP (<400 ng/mL, ≥400 ng/mL)**	1.069 (0.349–3.274)	0.907
**Child–Pugh class (A, B)**	0.781 (0.239–2.556)	0.683
**Largest tumor size (< 5 cm, ≥ 5cm)**	1.846 (0.434–7.850)	0.406
**BCLC stage (A, B, C)**	1.810 (0.547–5.992)	0.331
**Liver tumor number (<3, ≥3)**	0.550 (0.171–1.771)	0.316
**Macro vascular invasion (+, −)**	1.444 (0.459–4.537)	0.530
**Extrahepatic metastasis (+, −)**	1.443 (0.458–4.536)	0.529
**cTACE treatment (+, −)**	0.225 (0.062–0.814)	0.023*

*p < 0.05.

### cTACE prolonged the survival of HCC patients with PD-1 inhibitors and TKIs

**Figure 4:**
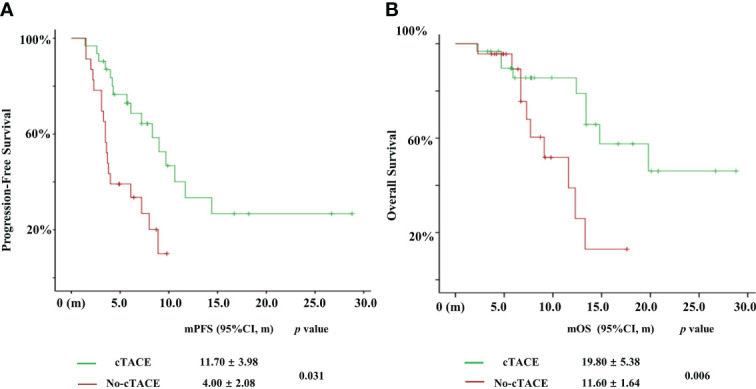
Kaplan-Meier analysis of PFS **(A)** and OS **(B)** in both groups.

The median PFS in the cTACE group was 11.70 months (95% confidence interval [CI], 3.90–19.50) compared to 4.00 months (95% CI, 0.00–8.07) in the no-cTACE group (**Figure 4A**, *P* = 0.031). The median OS in the cTACE group was estimated as 19.8 months (95% CI, 9.26–30.34) compared to 11.6 months (95% CI, 8.39–14.81) in the no-cTACE group (**Figure 4B**, *P* = 0.006). Similar results were found in the cohort without Lenvatinib bearing patients (**Figure S1**, mPFS was 16.40 vs. 3.70, *P* = 0.028; mOS was 21.00 vs. 11.60, *P* = 0.017). Cox regression was used to show the independent risk factors for PFS and OS (**Tables 4** and **5**). cTACE treatment and ECOG performance status (< 2) were beneficial for both PFS (hazard ratio [HR], 2.466; *P* = 0.019 and HR, 2.719; *P* = 0.008) and OS (HR, 3.471; *P* = 0.013 and HR, 2.392; *P* = 0.037). Patients with a poor ECOG performance status (= 2) also had a longer PFS (11.70 vs. 2.30 months) and OS (13.40 vs. 7.70 months) in the cTACE group (n = 9) compared to the no-cTACE group (n = 8), although there was no significant difference between the 2 groups (**Figure S2**, *P* > 0.05). Thirty-one patients reached PFS event until Mar 2022 and eight of them suffered without any other treatment. The therapeutic schedule post progression included Regorafenib, local treatment plus Regorafenib or Sintilimab (a PD-1 inhibitor from Innovent Biological company, Suzhou, China) plus Bevacizumab (anti-VEGF antibody from Roche company, Shanghai, China), and the constituent ratio of sequential treatment had no difference between cTACE and no-cTACE group (**Table S2**), indicating the choice of second-line was even and played subtle effect on the survival in two groups.

**Table 4:** Potential risk factors for PFS across the entire cohort.

Risk factors	Univariable	*P* value	Multivariable	*P* value
	HR (95% CI)		Adjusted HR (95% CI)	
**Sex (Male, Female)**	0.510 (0.154–1.693)	0.272		
**Age (< 60 y, ≥ 60 y)**	1.260 (0.587–2.702)	0.553		
**ECOG performance status (0-1, 2)**	2.428 (1.172–5.032)	0.017#	2.719 (1.293–5.721)	0.008*
**HBs–Ag (+, −)**	0.410 (0.141–1.189)	0.101		
**AFP (<400 ng/mL, ≥400 ng/mL)**	1.439 (0.707–2.931)	0.316		
**Child–Pugh class (A, B)**	0.973 (0.465–2.034)	0.941		
**Largest tumor size (< 5 cm, ≥ 5cm)**	1.117 (0.453–2.752)	0.810		
**BCLC stage (A, B, C)**	1.156 (0.585–2.284)	0.676		
**Liver tumor number (<3, ≥3)**	0.634 (0.306–1.312)	0.219		
**Macro vascular invasion (+, −)**	0.671 (0.331–1.359)	0.268		
**Extrahepatic metastasis (+, −)**	0.837 (0.412–1.701)	0.623		
**cTACE treatment (+, −)**	2.195 (1.051–4.587)	0.037#	2.466 (1.169–5.243)	0.019*

#p < 0.1, *p < 0.05.

**Table 5:** Potential risk factors for OS across the entire cohort.

Risk factors	Univariable	*P* value	Multivariable	*P* value
	HR (95% CI)		Adjusted HR (95% CI)	
**Sex (Male, Female)**	0.481 (0.140–1.650)	0.245		
**Age (< 60 y, ≥ 60 y)**	1.500 (0.631–3.565)	0.358		
**ECOG performance status (0-1, 2)**	2.575 (1.143–5.801)	0.022#	2.392 (1.055–5.426)	0.037*
**HBs–Ag (+, −)**	0.391 (0.112–1.360)	0.140		
**AFP (<400 ng/mL, ≥400 ng/mL)**	1.173 (0.526–2.612)	0.697		
**Child–Pugh class (A, B)**	0.731 (0.310–1.724)	0.474		
**Largest tumor size (< 5 cm, ≥ 5cm)**	1.119 (0.375–3.340)	0.841		
**BCLC stage (A, B, C)**	1.557 (0.526–4.611)	0.424		
**Liver tumor number (<3, ≥3)**	0.538 (0.237–1.219)	0.138		
**Macro vascular invasion (+, −)**	0.645 (0.285–1.459)	0.292		
**Extrahepatic metastasis (+, −)**	1.357 (0.595–3.091)	0.468		
**cTACE treatment (+, −)**	3.665 (1.384–9.709)	0.009#	3.471 (1.295–9.301)	0.013*

Note: #p < 0.1, * p< 0.05.

### Safety of cTACE sequentially administered with PD-1 inhibitors and TKIs

Different grades of TRAEs during treatment were compared between the groups as shown in **Table 6**. All types of serious TRAEs showed no difference between the cTACE and no-cTACE groups. Hepatic dysfunction was the most common TRAE in the whole cohort; there were more patients with all grades of elevated alanine aminotransferase and aspartic aminotransferase levels in the cTACE group than in the no-cTACE group (87.1% vs. 30.4% and 100.0% vs. 78.3%, respectively). Hematological toxicity (up to 77.4%), rash (up to 58.1%), and hand–foot syndrome (up to 52.2%) were the top 3 TRAEs expected for hepatic dysfunction, and no difference was found between the groups. Gastrointestinal reaction including diarrhea and poor appetite was also common post systemic treatment. The incidence of hypothyroidism was from 32.3% to 34.8%, which was consistent with other reported. Reactive cutaneous capillary endothelial proliferation (RCCEP, a specific skin AE of Camrelizumab) in our study was not common as other reported, for the anti-angiogenesis treatment was used at the same time. No patients suspended treatment due to adverse reactions. The relationship between TRAEs and ORR was analyzed in **Table 7**. Fourteen patients suffered with grade 1-2 of rash in 21 patients with CR or PR, while only 10 patients suffered with rash in 33 patients with SD or PD, identifying rash as an independent event for treatment effect prediction.

**Table 6:** TRAEs during treatment in both groups.

Clinic features	All grades of TRAEs		*P* value	Grade 3–4 of TRAEs		*P* value
	cTACEn = 31	No-cTACEn = 23		cTACEn = 31	No-cTACEn = 23	
**Leucopenia**	7 (22.6%)	3 (13.0%)	0.591	1 (3.2%)	0 (0.0%)	1.000
**Thrombocytopenia**	15 (48.4%)	13 (56.5%)	0.554	0 (0.0%)	0 (0.0%)	---
**Lymphopenia**	24 (77.4%)	12 (52.2%)	0.052	4 (12.9%)	3 (13.0%)	1.000
**Anemia**	13 (41.9%)	11 (47.8%)	0.667	0 (0.0%)	0 (0.0%)	---
**Elevated ALT**	27 (87.1%)	7 (30.4%)	0.000*	4 (12.9%)	2 (8.7%)	0.961
**Elevated AST**	31 (100.0%)	18 (78.3%)	0.024*	2 (6.5%)	3 (13.0%)	0.725
**Elevated GGT**	29 (93.5%)	18 (78.3%)	0.213	4 (12.9%)	3 (13.0%)	1.000
**Elevated TBIL**	20 (64.5%)	9 (39.1%)	0.064	0 (0.0%)	2 (8.7%)	0.345
**Hypothyroidism**	10 (32.3%)	8 (34.8%)	0.846	2 (6.5%)	2 (8.7%)	1.000
**Hand-foot syndrome**	15 (48.4%)	12 (52.2%)	0.783	3 (9.7%)	2 (8.7%)	1.000
**Rash**	18 (58.1%)	8 (34.8%)	0.090	1(3.2%)	1 (4.3%)	1.000
**RCCEP**	3 (9.7%)	2 (8.7%)	1.000	0 (0.0%)	0 (0.0%)	---
**Urine protein**	5 (16.1%)	3 (13.0%)	1.000	0 (0.0%)	0 (0.0%)	---
**Muscle soreness**	3 (9.7%)	3 (13.0%)	1.000	0 (0.0%)	0 (0.0%)	---
**Diarrhea**	7 (22.6%)	5 (21.7%)	0.941	0 (0.0%)	0 (0.0%)	---
**Poor appetite**	10 (32.3%)	4 (17.4%)	0.358	0 (0.0%)	0 (0.0%)	---
**Gingival bleeding**	4 (12.9%)	3 (13.0%)	1.000	0 (0.0%)	0 (0.0%)	---

*p< 0.05.

ALT, alanine aminotransferase; AST, aspartic aminotransferase; GGT, γ- glutamyl transpeptidase; TBIL, total bilirubin; RCCEP, reactive cutaneous capillary endothelial proliferation.

**Table 7:** Logistic analysis of adverse events for ORR during treatment in all patients.

Risk factors	Univariable	*P* value	Multivariable	*P* value
	HR (95% CI)		Adjusted HR (95% CI)	
**Leucopenia**	0.461 (0.036–5.890)	0.551		
**Thrombocytopenia**	0.677 (0.086–5.307)	0.710		
**Lymphopenia**	0.493 (0.080–3.033)	0.445		
**Anemia**	0.335 (0.060–2.084)	0.251		
**Elevated ALT**	2.420 (0.398–14.734)	0.338		
**Elevated AST**	0.162 (0.007–3.629)	0.251		
**Elevated GGT**	3.355 (0.195–57.720)	0.404		
**Elevated TBIL**	0.936 (0.145–6.052)	0.945		
**Hypothyroidism**	0.236 (0.029–1.889)	0.174		
**Hand–foot syndrome**	1.004 (0.152–6.618)	0.997		
**Rash**	9.420 (1.213–73.189)	0.032#	4.382 (1.297–14.803)	0.017*
**RCCEP**	8.236 (0.186–364.788)	0.276		
**Urine protein**	0.053 (0.004–0.645)	0.021#		
**Muscle soreness**	0.655 (0.019–22.765)	0.815		
**Diarrhea**	0.145 (0.013–1.611)	0.116		
**Poor appetite**	0.259 (0.029–2.322)	0.228		
**Gingival bleeding**	5.497 (0.311–97.185)	0.245		

#p < 0.1, *p < 0.05.

ALT, alanine aminotransferase; AST, aspartic aminotransferase; GGT, γ- glutamyl transpeptidase; TBIL, total bilirubin; RCCEP, reactive cutaneous capillary endothelial proliferation.

## Discussion

TACE plays an important role and is recommended to be combined with other methods to improve the long-term curative effect in advanced HCC (23). Due to its high heterogeneity, the recommended combination therapeutic method and timing are still being investigated by several clinical trials as referred above. In this study, we discussed the efficacy and safety of TACE combined with systemic therapy for advanced HCC patients in China.

Cao et al. (24) examined TACE combined with Sintilimab, and Lenvatinib in 60 unresectable HCC patients and reported the median PFS and OS were 13.3 and 23.6 months, respectively, suggesting the efficacy of this combination. However, the Lenvatinib was administered pre-TACE and then sequentially with Sintilimab, which was also performed in the same order in another report (25). Moreover, 2 studies investigated the efficacy of DEB-TACE sequentially with Camrelizumab plus Apatinib (AC) within 1 week in HCC patients (26, 27), but 1 study retrospectively compared AC combined with DEB-TACE or not. Here, the median OS was 24.8 months in the DEB-TACE with the AC group versus 13.1 months in the AC group. In our study, cTACE, which costs less than DEB-TACE, was used and sequentially combined with Camrelizumab plus TKIs. Patients with TACE treatment obtained a better ORR and longer OS compared to those in the no-TACE group, no matter the order of TKIs and TACE, indicating that TACE treatment is an independent risk factor for the prognosis of advanced HCC patients. The ORR in our study was evaluated after two cycles and four cycles of systemic treatment, reflecting a real long-term efficacy of the ICI combination. TACE induced a fast clinical remission of the tumor; further combination with immunotherapy prolonged the long-term benefit of TACE in patients. Nevertheless, the studies mentioned above-excluded patients with poor ECOG performance statuses and high Child–Pugh scores, while our study included 17 (33.5%) patients with an ECOG performance status of 2 and 7 (12.9%) with a Child–Pugh score > 7 points, leading to a shorter OS in our study. However, patients with a poor ECOG performance status also benefited from cTACE treatment, although without a statistically significant difference.

In addition, HBV infection is a primary etiological factor for HCC and exerts complex biological effects on the tumor microenvironment, resulting in immunosuppression by the downregulation of T-cells (28). CheckMate 040 data showed that the ORR of HBV-positive patients with nivolumab was lower than that of HBV-negative patients (29, 30). Our population was approximately 90% HBV cases, and the ORR in the TACE group was improved by about 30% compared to that in the no-TACE group, indicating that TACE combination therapy further activated the immune response in HBV-related HCC based on ICIs plus TKIs. Given the high rate of objective release, this combination mode can also be recommended as a neoadjuvant transformation therapy in some unresectable patients (31).

In contrast to the above, no difference in serious adverse events was investigated between the TACE and no-TACE groups, which was the same result as in other reports (25, 26), indicating the safety of this therapeutic combination. Furthermore, skin rash was reported as a common adverse event with both PD-1 inhibitors and TKIs as tumor therapy, so the treatment response might be related to rash (32), which is consistent with our results.

There are also several limitations of this study. First, this was a retrospective study with limited participants, although its conclusion is consistent with those of other reports published recently (25, 26). Second, it considered three different TKI medications used in this study (Sorafenib, Lenvatinib, and Apatinib), which had similar targets and supported this combination with PD-1 inhibitor theoretically. The bias of different medications was inevitable and further subgroup study with more cases for each medication should be performed to confirm our results. Finally, the interval of sequential systemic therapy was up to 8 weeks post-TACE, as determined by the physical condition and tumor burden of the patients, which might be another factor leading to the shorter survival in our study compared to other reports.

Based on these results, we reported a clinical combined treatment option with a definite curative effect and durable safety in HCC patients. Irrespective of the application order of TKIs, cTACE offered additional benefits above ICIs and TKIs only in advanced unresectable HCC patients. This option compensated for the deficiency of the long-term efficacy of TACE without increasing adverse events. More prospective clinical study data should be collected to verify this approach in the future.

## Data availability statement

The raw data supporting the conclusions of this article will be made available by the authors, without undue reservation.

## Ethics statement

The studies involving human participants were reviewed and approved by First Affiliated Hospital of Gannan Medical University. Written informed consent for participation was not required for this study in accordance with the national legislation and the institutional requirements.

## Author contributions

ZG designed the study, organized the data and wrote the manuscript. HZ collected the information of patients and contributed to writing the manuscript. XZ edited the manuscript for important intellectual content. LH performed statistical analysis. XW contributed to writing the manuscript. HS revised the manuscript. LY designed the study. YQ designed the study and evaluated the efficacy of treatment. FT designed the study and performed statistical analysis. All authors contributed to the article and approved the submitted version.

## Funding

This study was supported by the National Nature Science Foundation of China (# 81860440), the Jiangxi Provincial Natural Science Foundation (# 20202BAB206047). Shenzhen Key Laboratory Foundation (ZDSYS20200811143757022). Sanming Project of Medicine in Shenzhen (SZSM202111004).

## Conflict of interest

The authors declare that the research was conducted in the absence of any commercial or financial relationships that could be construed as a potential conflict of interest.

## Publisher’s note

All claims expressed in this article are solely those of the authors and do not necessarily represent those of their affiliated organizations, or those of the publisher, the editors and the reviewers. Any product that may be evaluated in this article, or claim that may be made by its manufacturer, is not guaranteed or endorsed by the publisher.
