# Supplementary figures and images for: The efficacy and safety of conventional transcatheter arterial chemoembolization combined with PD-1 inhibitor and anti-angiogenesis tyrosine kinase inhibitor treatment for patients with unresectable hepatocellular carcinoma: a real-world comparative study
Source: Front Oncol. 2022 Sep 29;12:941068. doi: 10.3389/fonc.2022.941068 (PMC9558003; doi:10.3389/fonc.2022.941068)

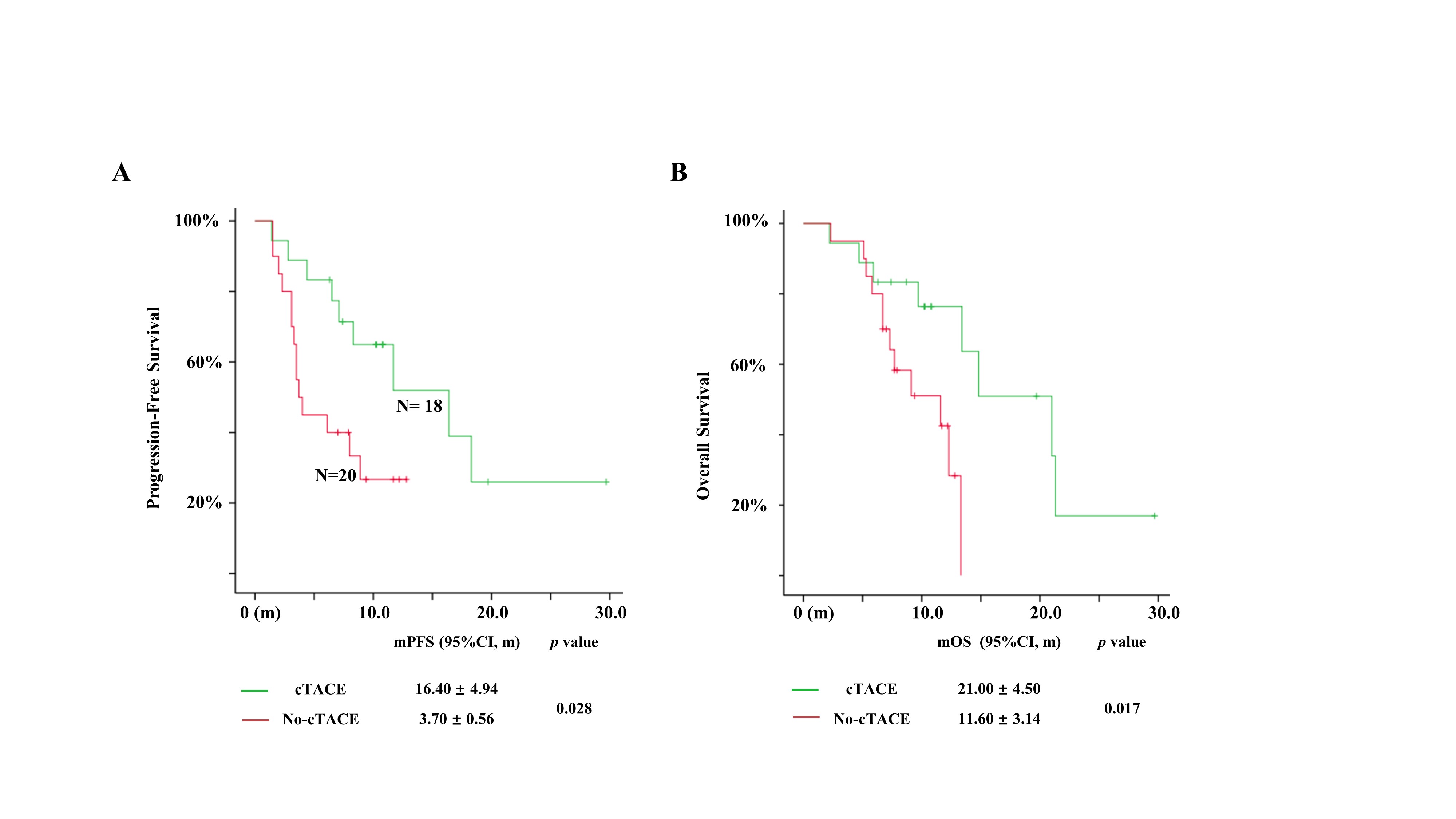

Supplement: Supplementary file 1 [file Image_1.jpeg]

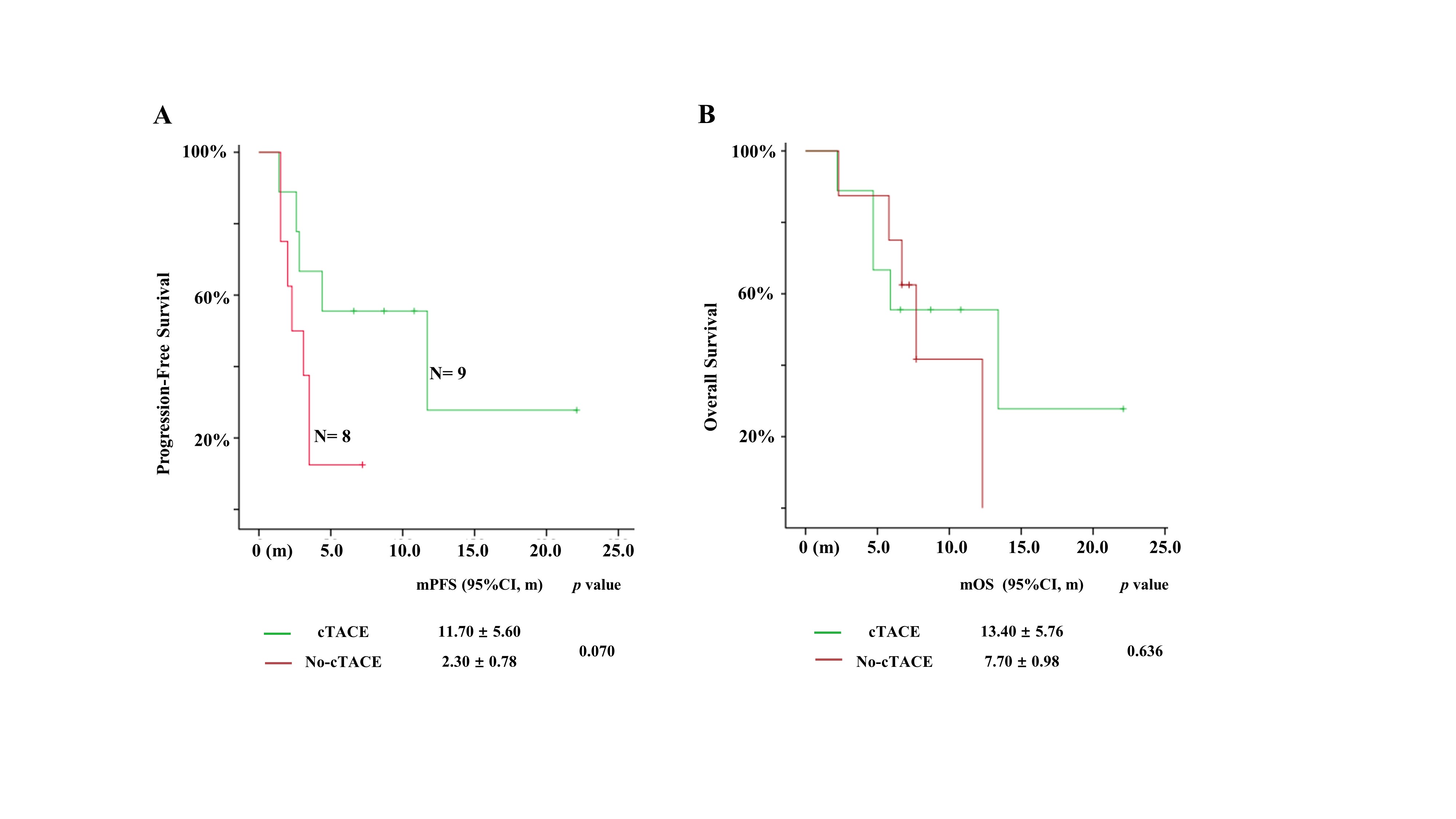

Supplement: Supplementary file 2 [file Image_2.jpeg]
