# Supplementary material for: The efficacy and safety of conventional transcatheter arterial chemoembolization combined with PD-1 inhibitor and anti-angiogenesis tyrosine kinase inhibitor treatment for patients with unresectable hepatocellular carcinoma: a real-world comparative study
Source: Front Oncol. 2022 Sep 29;12:941068. doi: 10.3389/fonc.2022.941068 (PMC9558003; doi:10.3389/fonc.2022.941068)
Supplement: Supplementary file 3 [file DataSheet_1.docx]

Table S1a. Efficacy of HCC patients in two cohort excluded with Lenvatinib bearing (with two cycles of ICI).

| Group  Efficacy | cTACE  (n = 18) | No-cTACE  (n = 20) | *P* value |
| --- | --- | --- | --- |
| PR  SD  PD | 9 (50.0%)  7 (38.9%)  2 (11.1%) | 4 (20.0%)  10 (50.0%)  6 (30.0%) | 0.042* |
| ORR (CR + PR)  (+)  (−) | 9 (50.0%)  9 (50.0%) | 4 (20.0%)  16 (80.0%) | 0.087 |
| DCR (CR + PR + SD)  (+)  (−) | 16 (88.9%)  2 (11.1%) | 14 (70.0%)  6 (30.0%) | 0.238 |

Table S1b. Efficacy of HCC patients in two cohort excluded with Lenvatinib bearing (with four cycles of ICI).

| Group  Efficacy | cTACE  (n = 18) | No-cTACE  (n = 20) | *P* value |
| --- | --- | --- | --- |
| CR  PR  SD  PD | 1 (5.6%)  9 (50.0%)  6 (33.3%)  2 (11.1%) | 0 (0.0%)  5 (25.0%)  8 (40.0%)  7 (35.0%) | --- |
| ORR (CR + PR)  (+)  (−) | 10 (55.6%)  8 (44.4%) | 5 (25.0%)  15 (75.0%) | 0.096 |
| DCR (CR + PR + SD)  (+)  (−) | 16 (83.3%)  2 (16.7%) | 13 (65.0%)  7 (35.0%) | 0.130 |

Table S2. The treatment difference in two groups post progression until Mar. 2022.

| Subsequential  treatment  post PD  Group | No treatment | Regorafenib | Local treatment + Regorafenib | Sintilimab +Bevacizumab | Total | Adjusted p value |
| --- | --- | --- | --- | --- | --- | --- |
| cTACE | 3 | 4 | 5 | 4 | 16 | 0.226 |
| No-cTACE | 5 | 4 | 2 | 4 | 15 |  |
